# Expression of Intracellular Interferon-Alpha Confers Antiviral Properties in Transfected Bovine Fetal Fibroblasts and Does Not Affect the Full Development of SCNT Embryos

**DOI:** 10.1371/journal.pone.0094444

**Published:** 2014-07-08

**Authors:** Dawei Yu, Shoufeng Zhang, Weihua Du, Jinxia Zhang, Zongxing Fan, Haisheng Hao, Yan Liu, Xueming Zhao, Tong Qin, Huabin Zhu

**Affiliations:** 1 Embryo Biotechnology and Reproduction Laboratory, Institute of Animal Science, Chinese Academy of Agricultural Sciences, Beijing, China; 2 Institute of Military Veterinary, Academy of Military Medical Science, Changchun, China; 3 State Key Laboratories of Agrobiotechnology, College of Biological Science, China Agricultural University, Beijing, China; The University of Melbourne, Australia

## Abstract

Foot-and-mouth disease, one of the most significant diseases of dairy herds, has substantial effects on farm economics, and currently, disease control measures are limited. In this study, we constructed a vector with a human *interferon-α* (*hIFN-α*) (without secretory signal sequence) gene cassette containing the immediate early promoter of human cytomegalovirus. Stably transfected bovine fetal fibroblasts were obtained by G418 selection, and *hIFN-α* transgenic embryos were produced by somatic cell nuclear transfer (SCNT). Forty-six transgenic embryos were transplanted into surrogate cows, and five cows (10.9%) became pregnant. Two male cloned calves were born. Expression of hIFN-α was detected in transfected bovine fetal fibroblasts, transgenic SCNT embryos, and different tissues from a transgenic SCNT calf at two days old. In transfected bovine fetal fibroblasts, expression of intracellular IFN-α induced resistance to vesicular stomatitis virus infection, increased apoptosis, and induced the expression of double-stranded RNA-activated protein kinase gene (*PKR*) and the 2′-5′-oligoadenylate synthetase gene (2′-5′ *OAS*), which are *IFN*-inducible genes with antiviral activity. Analysis by qRT-PCR showed that the mRNA expression levels of *PKR*, *2′-5′ OAS*, and *P53* were significantly increased in wild-type bovine fetal fibroblasts stimulated with extracellular recombinant human IFN-α-2b, showing that intracellular IFN-α induces biological functions similar to extracellular IFN-α. In conclusion, expression of intracellular hIFN-α conferred antiviral properties in transfected bovine fetal fibroblasts and did not significantly affect the full development of SCNT embryos. Thus, *IFN-α* transgenic technology may provide a revolutionary way to achieve elite breeding of livestock.

## Introduction

Transgenic technology enables the introduction of exogenous genes into animal genomes and provides a revolutionary way to achieve elite breeding of livestock. The main applications of transgenic technology in livestock breeding include improving their disease resistance, carcass composition, lactational performance, wool production, growth rate, and reproductive performance, as well as reducing their environmental impact [1].

Our efforts have focused on developing foot-and-mouth disease (FMD) resistance in dairy cattle using transgenic somatic cell nuclear transfer (SCNT) technology. FMD is a highly contagious vesicular disease of cloven-hoofed animals [2]. Outbreaks of FMD can have severe economic and social consequences that result in the loss of billions of dollars ($US) in direct and indirect costs, as well as the slaughter of millions of animals [3]. Current vaccines and disease-control measures to eliminate FMD have many drawbacks [4]. Several new strategies, such as RNA interference [5], [6], have been developed to control FMD, but few reports have detailed transgenic livestock strategies [1].

Evidence suggests that expression of exogenous IFN-α in livestock confers resistance to FMDV infection [2], [7]. Interferons (IFNs) are widely expressed cytokines that have potent antiviral and growth-inhibitory effects; they are the first line of defense against virus infections [8], [9]. However, several reports indicate that side effects are associated with over-expression of secreted IFN-α in animal models, such as disrupted spermatogenesis in male transgenic mice [10], [11].

In this study, cloned transgenic cattle containing IFN-α were generated to produce FMDV-resistant cattle. A secretory signal sequence of IFN-α was deleted to verify whether intracellular expression of IFN-α has side effects in transgenic cattle. We hypothesized that IFN-α without the secretory signal sequence would elicit the same biologic response as the secreted counterpart, but would not be secreted in transgenic SCNT embryos, would not trigger a signal transduction pathway in transgenic SCNT embryos and between pre-implant transgenic SCNT embryos and endometrial cells [12], and would have reduced toxicity to neighboring tissues [13].

To produce transgenic SCNT cattle that can resist FMDV, we constructed a vector with a human *IFN-α* gene cassette containing the immediate early promoter of human cytomegalovirus (HCMV). Transfected bovine fetal fibroblasts and transgenic SCNT embryos were obtained. After embryo transfer, transgenic cattle containing IFN-α were born at full term.

## Materials and Methods

### Animals

Cows were from the farm of the Chinese Academy of Agricultural Sciences, Beijing, China. They were allowed access to feed and water *ad libitum* under normal conditions. Experiments were performed according to the Regulations for the Administration of Affairs Concerning Experimental Animals (Ministry of Science and Technology, China, revised in June 2004) and approved by the Institutional Animal Care and Use Committee Chinese Academy of Agricultural Sciences, Beijing, China (Permit Number: IAS10012).

### Study design

In this study, a vector with a human *IFN-α* (*hIFN-α*) (without secretory signal sequence) gene cassette containing the immediate early promoter of HCMV was constructed. Stably transfected bovine fetal fibroblasts were obtained by G418 selection, and expression of intracellular hIFN-α, resistance to vesicular stomatitis virus infection, apoptosis, and expression of the genes *PKR*, *2′-5′ OAS* and *P53* were investigated.

Transgenic embryos were produced by SCNT. Transgenic SCNT embryos were transplanted into surrogate cows. Expression of intracellular hIFN-α was detected in the transgenic SCNT embryos and different tissues from a transgenic SCNT calf.

### Construction of plasmid vector

A new plasmid, pIRESneo-*IFN*-bCP-*LFCIN B*-*EGFP*, was constructed. pIRES1-neo (GenBank Acc. No. U89673; Cat.no. 6060, Clontech Laboratories, Palo Alto, CA, USA) was used as the basal plasmid for vector construction. The *hIFN-α* gene (GenBank Acc. no. X66186.1) was cloned into the vector at the *Eco*RV site. A mammary-gland-tissue-specific expression sequence, including the *lactoferricin B* (*LFCIN B*) gene (GenBank Acc. no. L08604) and goat β-casein promoter sequence (GenBank Acc. no. AY311384) [14], [15] was subcloned into the vector at *Bst*1107I; this can direct the expression of a foreign gene in the lactating mammary gland. The HCMV promoter sequence and *EGFP* gene were cloned into the vector at *Xho*I, forming pIRESneo-*IFN*-bCP-*LFCIN B*-*EGFP* (Fig. S1). The plasmid pEGFP-N1 (Clontech) was used as the control vector.

### Transfection of vector into bovine fetal fibroblasts

Bovine fetal fibroblasts were obtained from a fetus of a Holstein cow slaughtered 40 days after artificial insemination. Fibroblasts were isolated from a skin biopsy and cultured in DMEM (Gibco, Life Technology, Carlsbad, CA, USA) supplemented with 10% fetal bovine serum (Gibco, Life Technology) in a 37.5°C humidified incubator with 5% CO_2_.

Bovine fetal fibroblasts were transfected with the vector using an Amaxa Nucleofector (Lonza, Cologne, Germany), according to the manufacturer's protocol [16]. Forty-eight hours after transfection the cells were split 1∶16 and neomycin (Sigma, St. Louis, MO, USA) was added to a final concentration of 500 µg/mL. After 2 weeks, resistant colonies were obtained and stably transfected cells were established by expansion culture. Integration of *hIFN-α* DNA was verified by polymerase chain reaction (PCR) using a forward primer binding to the HCMV sequence (5′-GAA GTT GTT CGT GCT GAA AT-3′) and a reverse primer binding to the *hIFN-α* sequence (5′-GAG AAA GGC AAA GTG GAT GT-3′).

### Vesicular stomatitis virus infection

To harvest enough cells for vesicular stomatitis virus infection, the stably transfected bovine fetal fibroblasts were expanded for 10 passages in DMEM supplemented with 10% FBS (Gibco, Life Technology) and 300 ng neomycin/mL (Sigma). They were then plated at a density of 2.0×10^5^ cells/well in six-well, flat-bottomed plates. After incubation for 24 h under standard conditions, fibroblasts were infected with vesicular stomatitis virus (VSV) (provided by Professor Gang Li [Chinese Academy of Agricultural Sciences, Beijing, China]) at a multiplicity of infection (MOI) of 10 for 2 h. After culture in normal medium (without virus) for a further 72 h, culture medium was collected and centrifuged at 1,000 rpm/min for 5 min to remove cell debris. Cell viability was estimated using an apoptosis assay. Serial dilutions of each supernatant were added to non-transfected bovine fibroblasts in 96-well plates, and 24 h later the cytopathic effect (CPE) was observed using TE-2000 phase-contrast microscopy (Nikon, Tokyo, Japan). VSV in the supernatants was titrated according to the method of Kaerber [12].

### Somatic cell nuclear transfer

Transgenic SCNT embryos were produced using an improved method that combines micro-extrusion and zona-free fusion. The donor cells in the transfected group were the bovine fetal fibroblasts transfected stably with vector pIRESneo-*IFN*-bCP-*LFCIN B*-*EGFP* and cultured in DMEM supplemented with 10% FBS (Gibco, Life Technology) and 300 ng neomycin/mL (Sigma) for 8–10 passages. The donor cells in the control group were non-transfected bovine fetal fibroblasts and cultured in DMEM supplemented with 10% FBS (Gibco, Life Technology) for the same number of passages as above. First, matured oocytes were enucleated by micromanipulation; next, two zona-free enucleated oocytes were fused with single donor cell [17]; finally, reconstructed embryos were activated and cultured using the well-of-the-well (WOW) system [18]. On day 7, SCNT blastocysts cultured *in vitro* were transferred nonsurgically into Holstein heifers (15–18 months of age) at 6.5–7 d after estrus on a local farm (Beijing, China). A single embryo was transferred in bovine embryo transfer medium (Agtech, Manhattan, KS, USA) into a recipient uterine lumen ipsilateral to the corpus luteum. Recipient synchronization was achieved using a standard double-injection prostaglandin (Lutalyze; Pharmacia & Upjohn, Kalamazoo, MI, USA) treatment. Transgenic embryos were transferred into 46 Holstein heifers; non-transgenic embryos were transferred into 77 Holstein heifers. Pregnancies were diagnosed by rectal palpation between days 55 and 60.

### Real-time RT-PCR analyses

Quantitative real-time PCR (qRT-PCR) was used to detect the expression levels of the *hIFN*-*α*, *LFCIN B*, *PKR*, 2′-5′ *OAS*, and *P53* genes in transfected bovine fetal fibroblasts cultured for 10 passages.

For real-time RT-PCR analyses of cells, total RNA was extracted using TRIzol (Invitrogen, Life Technology), RNA purity was determined by measuring absorbance at 260 and 280 nm (A260/280), and RNA was reverse-transcribed using SUPERSCRIPT III (Invitrogen, Life Technology) as previously described [19]. The 7500 Fast Real-Time PCR System (Applied Biosystems, Foster City, CA, USA) was used to determine the level of cDNA using Taqman probe assays (Invitrogen, Life Technology) for the bovine *2′-5′ OAS*, *PKR*, *P53*, and *GAPDH* mRNAs in transfected cells. The program consisted of pre-denaturing at 95°C for 10 min; 40 cycles of denaturing at 95°C for 10 sec, annealing at 60°C for 30 sec and extension at 70°C for 45 sec. Data were normalized to the expression level of *GAPDH* in each sample. The primers and probe sequences are shown in Table 1. The Ct value was defined as the number of PCR cycles in which the fluorescence signal exceeded the detection threshold value. The 2^−ΔΔCt^ value, where ΔCt = Ct_Gene_ - Ct_GAPDH_ and ΔΔCt = ΔCt_treated_ - ΔCt _control_, was calculated to represent the relative mRNA expression of bovine *2′-5′ OAS*, *PKR*, *P53 and GAPDH* mRNA in antivirus analysis of transfected cells, non-transfected cells and wild type cells. SYBR green I (Invitrogen, Life Technology) was used for the *hIFN*-*α* and *LFCIN B* expression analysis. The ratio = (Etarget)^ΔCTtarget (control – sample)^/(Eref) ^ΔCTref (control – sample)^ was calculated to represent the relative mRNA expression of *hIFN*-*α* and *LFCIN B* in transfected bovine fetal fibroblasts, non-transfected cells, wild type cells and transgenic SCNT embryos [20].

**Table 1:** Primer and probe sequences.

Gene	Primers sequences (5′-3′)	Size (bp)	Probe sequences (5′-3′)
*hIFN-α*	F: GAT GCG TCG TAT CTC TCT GTT C	103	
	R: CGG GAT GGT TTC AGC TTT CT		
*LFCIN B*	F:TTG GAC TGT GTC TGG CTT TC	86	
	R: CCT CCT CAC ACA GGT GAT AGA		
*PKR*	F: AGT GCT GCG TGT GGT GAT GT	321	AAG AAA CTC TCC AGC AGT GAG TAC
	R: TGG AGA CAC GGA AGA GCT GTT		
*2′-5′ OAS*	F: AAA GAG GAA GCG GCG TGC	171	TCA GCT TCG TGC TCA GGT C
	R: CTC CTC AGG TTC TCG CAC TCT		
*P53*	F: AAC ACC AGC TCC TCT CCA CAG	115	AAG AAG AAA CCA CTG GAT GGA
	R: CCA AGG CAT CAT TCA GCT CTC		
*GAPDH*	F: AAG GCC ATC ACC ATC TTC CA	113	AGC GAG ATC CTG CCA ACA T
	R: CCA GCC TTC TCC ATG GTA GTG		

*Note*: *hIFN-α:* human *interferon*-α; *LFCIN B*: *Lactoferricin B*; *PKR*: double-stranded RNA-activated protein kinase; *2′-5′ OAS*: 2′-5′-oligoadenylate synthetase.

For real-time RT-PCR analyses of embryos, cDNAs of single embryos were synthesized with Cell to cDNA II kits (Ambion, Austin, TX, USA), and quantitative PCR was performed as previously described [21].

### Western blotting analysis

Tissues (liver, lung, spleen, heart, ear skin, muscle, stomach, intestine, colon, kidney) from a transgenic SCNT calf and transfected bovine fetal fibroblasts expanded for 10 passages were extracted in lysis buffer (1% NP-40, 150 mM NaCl, 5 mM EDTA, 50 mM NaF, 20 mM Tris–HCl, pH 7.4, 2 mM Na3VO4, 1 mM PMSF, and protease inhibitor cocktail). Proteins were quantified using a BCA Protein Assay kit. For western blot assays, equal amounts of protein (20 µg) were subjected to 12% SDS–PAGE and electrophoretically transferred to nitrocellulose (NC) membranes (0.45 µm, Millipore, Billerica, MA, USA). The non-specific sites on each blot were blocked with 3% BSA diluted in TBS with 0.05% Tween 20 (TBST) for 90 min. Proteins were detected using mouse monoclonal [9D3] antibody against IFNα-2b (Abcam, Cambridge, UK), purified mouse monoclonal antibody against LTF (Abgent, San Diego, CA, USA), or mouse monoclonal antibody against β-actin (Santa Cruz Biotechnology, Dallas, TX, USA) as primary antibody and horseradish peroxidase (HRP)-conjugated goat anti-mouse Ig G (Jackson ImmunoResearch, West Grove, PA, USA) as secondary antibody. Proteins were detected using an enhanced chemiluminescence (ECL) reagent (Millipore).

### Apoptosis assay

FITC-conjugated annexin V and propidium iodide (PI) staining was performed using a kit (Neobioscience, Shanghai, China). Briefly, after infection with VSV for 2 h and culture for a further 72 h in normal medium, the transfected bovine fetal fibroblasts were resuspended in binding buffer, washed twice for 3 min with PBS, and annexin V-FITC and PI were added. Following a 10 min incubation in the dark, samples were subjected to flow cytometric analysis with a FACScalibur flow cytometer (Becton Dickinson, San Jose, CA, USA) using Cell-Quest software [22]. Mechanically damaged cells were located in the upper left quadrant, late apoptotic or necrotic cells in the upper right quadrant, dual negative and normal cells in the lower left quadrant, and early apoptotic cells in the lower right quadrant of the flow cytometric dot plot. The total apoptotic rate was calculated as the late apoptotic rate + early apoptotic rate.

### Immunofluorescence and confocal microscopy

For staining of hIFN-α, transgenic SCNT blastocysts (generated using stably transfected bovine fetal fibroblasts as donors, followed by SCNT) (n = 10) cultured for 7 days *in vitro* were fixed and permeabilized in 2% paraformaldehyde and 0.5% Triton X-100 in PBS (pH 7.4) for at least 30 min at room temperature. Blastocysts were blocked in 0.1% Triton X-100 and 2% BSA-supplemented PBS for 1 h and incubated overnight at 4°C with 1∶200 V450-conjugated mouse anti-human IFN-α-2b (Becton Dickinson). After three washes in PBS, blastocysts were mounted on glass slides and examined with a Confocal Laser-Scanning Microscope (C-1, Nikon) under the Violet laser Em Max at 450 nm.

### Statistical analysis

Data in each experiment were expressed as means ± standard deviation (SD) from three independent experiments. Statistical analyses were performed using one-way analysis of variance (ANOVA) with Duncan's test for post hoc analysis using SAS version 8 (SAS Institute Inc., Cary, NC, USA). *P*<0.05 was considered significant.

## Results

### Expression of hIFN-α in transfected bovine fetal fibroblasts

**Figure 1:**
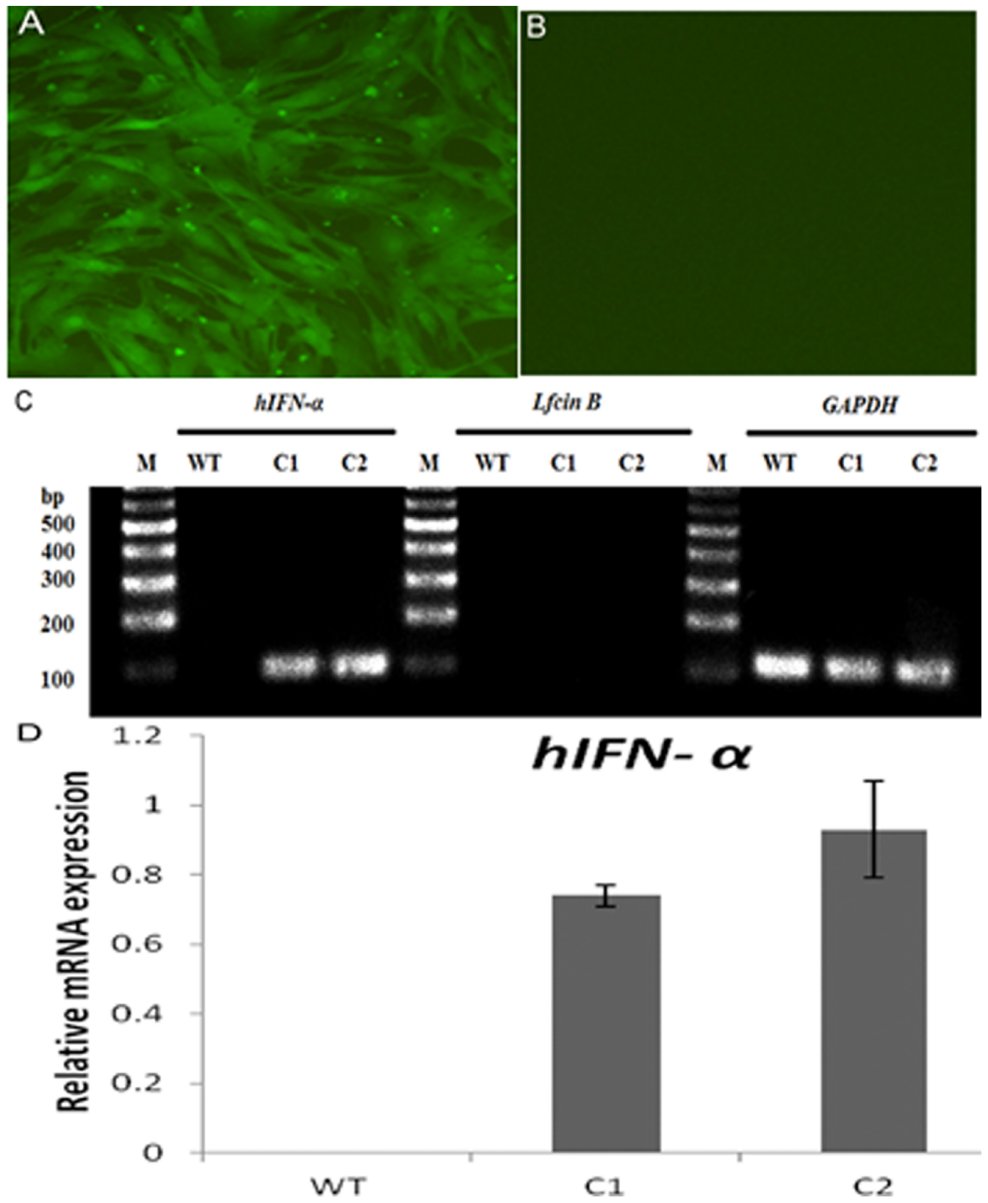
*hIFN-α* and *LFCIN B* gene expression in transfected bovine fetal fibroblasts. GFP was expressed in transfected bovine fetal fibroblasts (×200) (A), but not in wild-type bovine fetal fibroblasts (×200) (B). Green: green fluorescent protein (GFP). (C, D) *hIFN-α* and *LFCIN B* gene expression in transfected bovine fetal fibroblasts was analyzed using real-time RT-PCR, with results normalized to expression levels of *GAPDH*. WT, Wild-type bovine fetal fibroblasts; C1, transfected bovine fetal fibroblast clone 1; C2, transfected bovine fetal fibroblast clone 2. Relative mRNA expression levels are shown as means ± standard deviation (SD) from three independent experiments.

**Figure 2:**
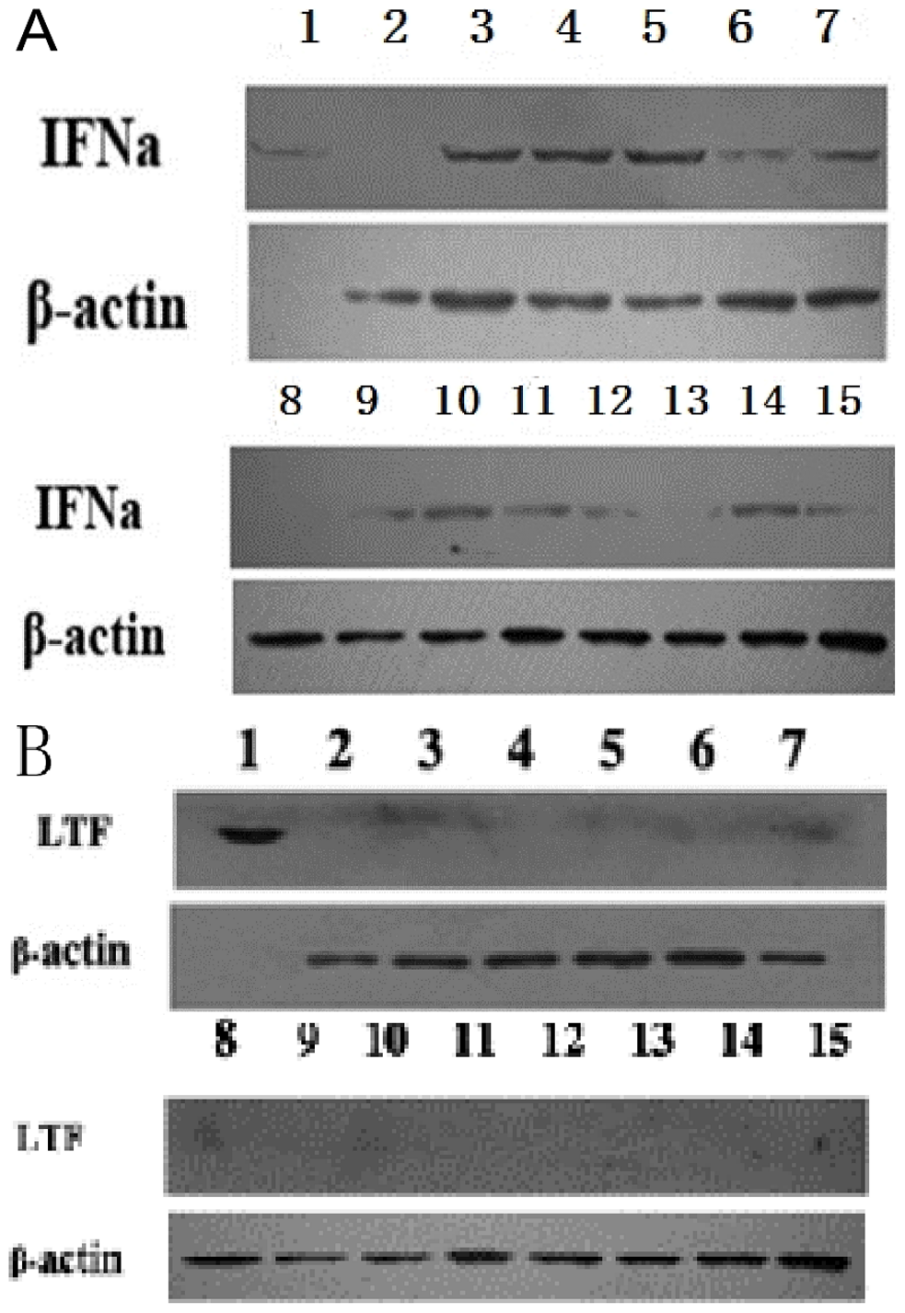
Detection of hIFN-α and LFCIN B (LTF) protein expression in transfected donor cells (transfected bovine fetal fibroblasts) and tissues from the transgenic calf generated by somatic cell nuclear transfer (SCNT). The hIFN-α (A) and LFCIN B (B) protein expression was determined by western blot. Lane 1 in (A), recombinant human IFN-α 2b protein as positive control. Lane 1 in (B), lactoferricin B as positive control; lanes 2 and 8, the same wild-type bovine fetal fibroblasts, as negative controls; lane 3, transfected bovine fetal fibroblast clone 1; lane 9, transfected bovine fetal fibroblast clone 2; lane 4, liver; lane 5, lung; lane 6, spleen; lane 7, heart; lane 10, ear skin; lane 11, muscle; lane 12: stomach; lane 13: intestine; lane 14: colon; lane 15: kidney.

The vector pIRESneo-*IFN*-bCP-*LFCIN B-EGFP* (Fig. S1) was constructed and used to transfect early-passage bovine fetal fibroblasts. Stable G418 resistant clones were derived. Under fluorescence microscopy, GFP expression was detected in stably transfected bovine fetal fibroblasts (Fig. 1A), but not in wild-type bovine fetal fibroblasts (Fig. 1B). RT-PCR analysis showed *hIFN-α* mRNA was expressed in stably transfected bovine fetal fibroblasts, but not in wild-type bovine fetal fibroblasts (Fig. 1D). *LFCIN B* mRNA was not expressed in transfected or wild-type bovine fetal fibroblasts (Fig. 1C). Total protein extracts from bovine fetal fibroblasts were analyzed by western blotting using monoclonal antibodies against hIFN-α-2b and LFCIN B, with hIFN-α protein expression was detected in transfected bovine fetal fibroblasts (Fig. 2A, lane 3, 9), but not in extracts from wild-type bovine fetal fibroblasts (Fig. 2A, lanes 2, 8). LFCIN B expression was not detected in transfected or wild-type bovine fetal fibroblasts (Fig. 2B, lanes 3, 9 and lanes 2, 8).

### Antiviral activity of hIFN-α in the transfected bovine fetal fibroblasts

**Figure 3:**
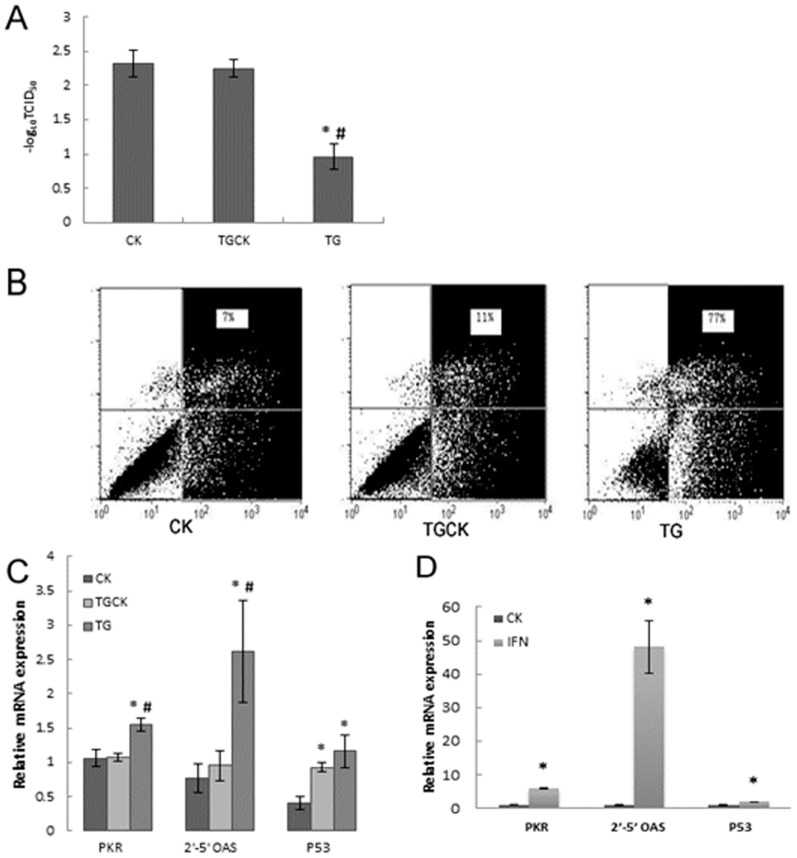
Antiviral activity and apoptosis mediated by hIFN-α in transfected bovine fetal fibroblasts. After VSV infection for 2(without virus) for a further 72 h. (A) Virus titres (TCID 50) in the supernatant of wild-type (CK), control vector (peGFP-N1 vector) transfected (TGCK), and *hIFN-α* transfected (TG) bovine fetal fibroblasts. (B) Apoptosis was determined by FITC-conjugated annexin V and propidium iodide (PI) staining. The y-axis is PI and the x-axis is FITC-annexin V. (C) The expression levels of *PKR*, *2′-5′ OAS*, and *P53* mRNA were analyzed using real-time RT-PCR with results normalized to the expression levels of *GAPDH*. (D) The expression levels of *PKR*, *2′-5′ OAS*, and *P53* mRNA in wild-type bovine fetal fibroblasts after 5×10^−5^ µg rhIFN-α-2b/mL was added to the culture medium for 2 h, analyzed by qRT-PCR with results normalized to expression levels of *GAPDH*. Data are shown as means ± SD from three independent experiments. *P<0.05 *vs.* CK group; #*P*<0.05 TG *vs.* TGCK. *PKR*, double-stranded RNA-activated protein kinase gene; *2′-5′ OAS*, 2′-5′-oligoadenylate synthetase gene.

To determine whether the expression of intracellular hIFN-α was sufficient to confer antiviral properties on transfected bovine fetal fibroblasts, fibroblasts were infected with VSV for 2 h. After culture in normal medium for a further 72 h, the concentration of VSV in the supernatants was titrated. Virus titer was markedly reduced in the supernatants of bovine fetal fibroblasts stably transfected with the *hIFN-α* gene compared with those in the supernatants of non-transfected and bovine fetal fibroblasts transfected with the control vector (pEGFP-N1 vector) (both *P*<0.05) (Fig. 3A). Virus titer was not significantly different between non-transfected and bovine fetal fibroblasts transfected with the control vector (*P*>0.05), indicating that the expression of hIFN-α conferred protection against viral infection on the transfected bovine fetal fibroblasts.

### Expression of intracellular hIFN-α increase transfected bovine fetal fibroblast apoptosis

After infection with VSV for 2 h and culture in normal medium for a further 72 h, cell apoptosis was estimated by staining with annexin V and PI, followed by flow cytometric analysis. Approximately 7% of non-transfected and 11% of transfected bovine fetal fibroblasts carrying the control vector underwent apoptosis, while expression of hIFN-α resulted in 77% apoptosis in infected transfected bovine fetal fibroblast (Fig. 3B).

### hIFN-α increases the mRNA expressions of antiviral genes *PKR*, 2′-5′ *OAS*, and *P53*

To test whether the expression of intracellular hIFN-α could trigger antiviral-related signaling events in *hIFN-α* transfected bovine fetal fibroblasts, the expression levels of *IFN*-inducible genes that stimulate antiviral activities, including *PKR*, 2′-5′ *OAS*, and *P53*, in transfected bovine fetal fibroblasts were analyzed using qRT-PCR. The results showed that the expression levels of these genes were significantly increased in *hIFN-α* transfected bovine fetal fibroblasts, compared with those in non-transfected bovine fetal fibroblasts (all *P*<0.05) (Fig. 3C). There was no significant difference in the expression levels of *PKR* and *2′-5′ OAS* between bovine fetal fibroblasts transfected with the control vector and non-transfected bovine fetal fibroblasts (both *P*>0.05). However, the expression of *P53* was significantly increased in bovine fetal fibroblasts transfected with the control vector. To confirm that intracellular *IFN-α* induced biological functions similar to extracellular *IFN-α*, as previously reported [12], [13], [23], qRT-PCR analysis was used to show that the levels of expression of *PKR*, *2′-5′ OAS*, and *P53* mRNA were significantly increased in wild-type bovine fetal fibroblasts stimulated with 5×10^−5^ µg extracellular recombinant human IFN-α-2b (rhIFN-α-2b) (Sigma)/mL for 2 h (all *P*<0.05) (Fig. 3D).

### Expression of hIFN-α in *hIFN-α* transgenic SCNT embryos

**Figure 4:**
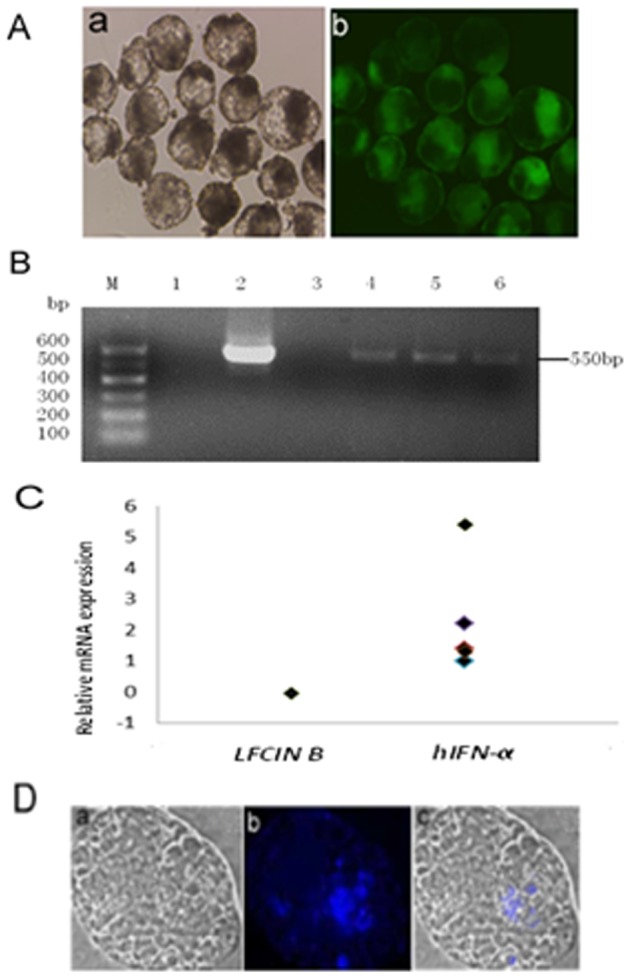
Detection of hIFN-α and LFCIN B expressed in transgenic SCNT embryos by PCR and immunofluorescence. (A) zona-free transgenic SCNT blastocyst (×100). Panel a, under light microscopy; Panel b, under fluorescence microscopy. Green: GFP. (B) PCR analysis of the *hIFN-α* gene in transgenic SCNT embryos. Lane M, 100 bp molecular weight marker; lane 1, water as template; lane 2, pIRSEneo-*IFN*-bCP-*LFCINB*-*EGFP* as template; lane 3, non-transgenic SCNT embryos DNA as template; lanes 4, 5, and 6, *hIFN-α* transgenic SCNT embryos. (C) Real-time RT-PCR analysis of mRNA expression levels of *hIFN-α* (n = 6) and *LFCIN B* (n = 6) in transgenic SCNT embryos, with results normalized to expression levels of *GAPDH*. (D) Immunofluorescence analysis of hIFN-α expressed in transgenic SCNT embryos (×200, n = 10). Transgenic SCNT blastocysts were stained with V450-conjugated antibody against hIFN-α-2b. Panel a, under light microscopy; Panel b, under fluorescence microscopy with Violet laser Em Max at 450 nm; Panel c, merged images, with blue showing V450 fluorescence.

Transgenic SCNT embryos were produced using an improved SCNT method that combines microextrusion and zona-free fusion, resulting in a higher fusion rate than microinjection (data not shown). Green fluorescent protein (GFP) was observed in transgenic SCNT embryos under fluorescence microscopy (Fig. 4A). The integration of foreign genes within the genome of the SCNT embryos was detected using PCR (Fig. 4B). Analysis by qRT-PCR showed that *hIFN-α* mRNA was expressed in the transgenic SCNT embryos, but that *LFCIN B* mRNA was not (Fig. 4C). IFN-α protein in the transgenic SCNT embryos was also expressed and detected by immunofluorescence (Fig. 4D). To test the potential effect of hIFN-α intracellular expression on the developmental competence of the transgenic SCNT embryos, early development of *hIFN-α* transgenic SCNT embryos and non-transgenic SCNT embryos was compared. No significant differences were found in cleavage rate, blastocyst rate (Table 2), or pregnancy outcomes (Table 3) between them after embryo transfer.

**Table 2:** Development of *hIFN-α* transgenic SCNT embryos and non-transgenic SCNT embryos.

Donor cells	No. of embryos reconstructed	Fusion rate (%)	Cleavage rate (%)	Blastocyst rate (%)
WT	248	94.80±1.11	95.09±0.91	34.22±0.91
IFNTG	285	96.04±1.52	93.35±2.01	33.78±6.95

*Note*: the data are shown as means ± standard deviation (SD). WT: wild-type bovine fetal fibroblasts; IFNTG: *hIFN-α* transfected bovine fetal fibroblasts.

**Table 3:** Pregnancies of recipients of transgenic SCNT embryos and non-transgenic SCNT embryos.

SCNT embryos	No. transplanted embryos	NO. pregnancy (%)	NO. live-born
NonTG	77	10 (13.0%)	2
IFNTG	46	5 (10.9%)	2

*Note:* NonTG: non-transgenic SCNT embryos; IFNTG: *hIFN-α* transgenic SCNT embryos.

### Analysis of hIFN-α expression in the SCNT calf

**Figure 5:**
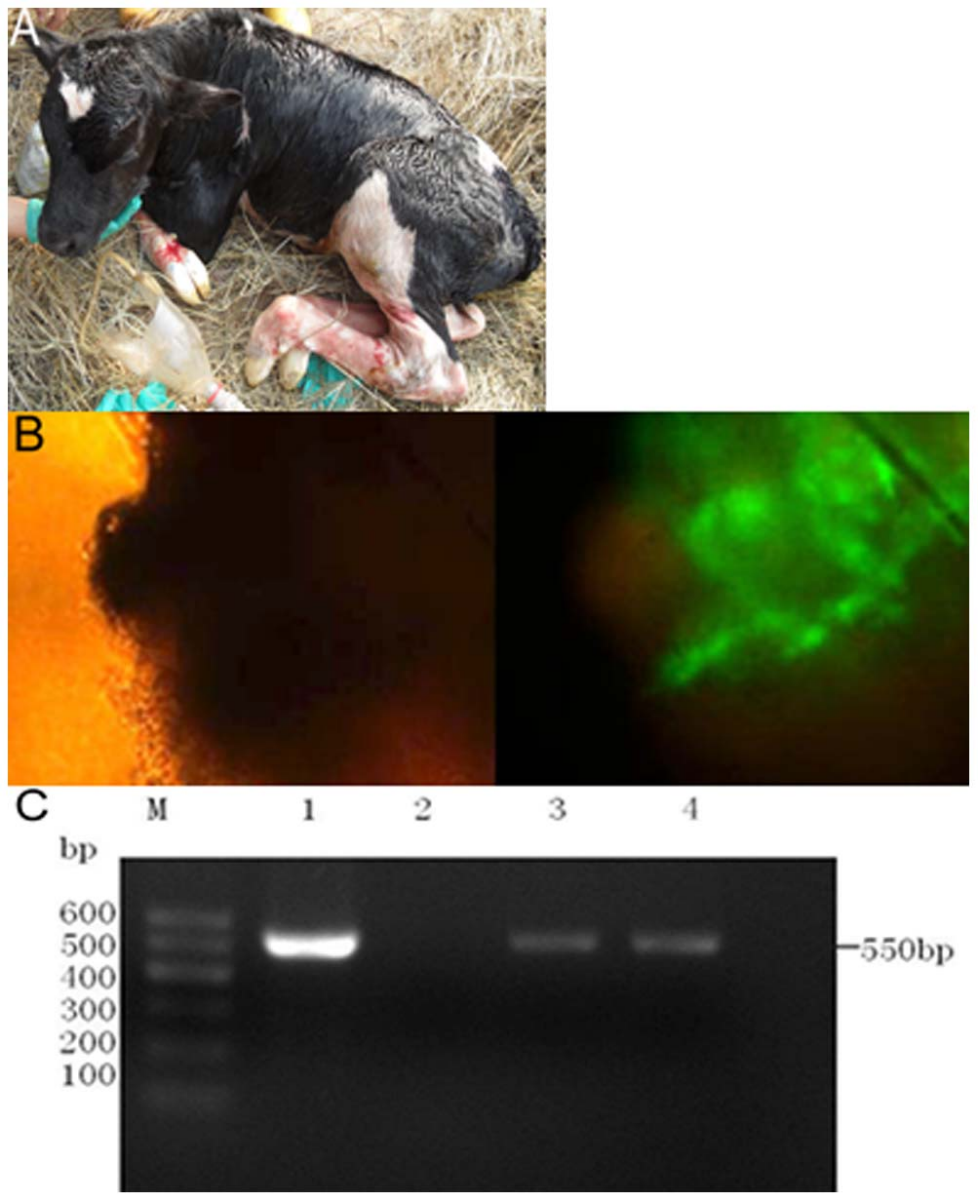
Detection of hIFN-α expression in the transgenic SCNT calf by PCR and GFP expression by fluorescence microscopy. (A) Live-born transgenic calf. (B) GFP expressed in ear skin from the transgenic SCNT calf that died at two days of age under light microscopy (×40) and under fluorescence microscopy (×40). Green: GFP. (C) PCR analysis of the *hIFN-α* gene expressed in the transgenic SCNT calf that died at two days of age. Lane M, 100 bp molecular weight marker; lane 1, pIRSEneo-*IFN*-bCP-*LFCIN B*-*EGFP* as template; lane 2, DNA from the wild-type calf as a template; lane 3, blood from the transgenic SCNT calf; lane 4, *funiculus umbilicalis* from the transgenic SCNT calf.

Forty-six transgenic SCNT embryos were transplanted into surrogate cows, and five cows (10.9%) became pregnant (Table 3). Two male cloned calves were born (Fig. 5A). One calf died of excessive bleeding due to umbilical cord hypogenesis at two days old. GFP was detected by fluorescence microscopy in the ear skin from the transgenic SCNT calf that died at two days of age (Fig. 5B). DNA samples from a wild-type calf and the transgenic SCNT calf that died at two days of age were screened by PCR using primers designed to amplify a 550-bp fragment in the gene cassette. Results showed that blood and the *funiculus umbilicalis* from the transgenic SCNT calf carried the *hIFN*-α gene (Fig. 5C). IFN-α was expressed in the different tissues (liver, lung, spleen, heart, ear skin, muscle, stomach, intestine, colon and kidney) of the transgenic SCNT calf that died at two days, as detected by western blotting (Fig. 2A, lanes 4–7, 10–15). However, LFCIN B was not detected in these tissues (Fig. 2B, lanes 4–7, 10–15).

## Discussion

In our preliminary study, we focused on biological activities of IFN-α in transfected fetal fibroblasts and transgenic SCNT embryos. We constructed a vector with a bovine *LFCIN B* gene cassette containing a goat β-casein regulatory sequence and a human *IFN-α* (without secretory signal sequence) gene cassette containing the immediate early promoter of HCMV, and hIFN-α was expressed in both transfected bovine fetal fibroblasts and transgenic SCNT embryos, whereas LFCIN B, which was regulated by the goat β-casein promoter was only expected to be expressed during lactation. Two male cloned transgenic calves were born and were not expected to express the *LFCIN B* gene.

To distinguish exogenous *IFN-α* from endogenous *IFN-α* possibly produced by bovine cells, *hIFN-α* was cloned into the vector. The hIFN-α significantly augmented the expression of IFN-inducible genes, which indicated that exogenous *hIFN-α* triggered the expected signal transduction pathway in bovine cells [24], even though the amino acid sequence of hIFN-α has only 60% identity with that of bovine IFN-α.

Expression of intracellular hIFN-α resulted in antiviral activity, increased apoptosis, and induced the expression of IFN-inducible genes in transfected fetal fibroblasts. Therefore, intracellular hIFN-α had activities similar to those of extracellular IFN-α in bovine cells. This finding is further supported by the observation that rhIFN-α-2b added to the culture medium of wild-type bovine fetal fibroblasts stimulated the expression of IFN-inducible genes. Several studies have indicated that exogenous IFN-α with a deleted secretory signal sequence (i.e., eliminated secretion), which cannot be secreted, exerts biological functions similar to those of extracellular IFN-α [12], [23]. Further studies are needed to elucidate the signaling pathway triggered by the intracellular ligand of IFN-α.

The mechanism of intracellular hIFN-α–mediated suppression of virus replication might have been apoptotic cell death induced by P53, which was significantly induced in *hIFN-α* transfected cells. Prompt induction of apoptosis of virus-infected cells via P53 activation is beneficial to the host in limiting virus replication. However, *P53* mRNA was significantly induced in fetal fibroblasts transfected with the control vector without the *hIFN-α* gene, and there was no significant difference compared to the *hIFN-α* transfected cell group. The induction of *P53* mRNA in transfected fetal fibroblasts containing the control vector might be caused by G418 selection pressure, under which cells that could not express enough neomycin resistance undergo apoptosis. This hypothesis was supported by our cell viability assay; the results showed that more cells went into apoptosis among cells transfected with the control vector than in infected wild-type bovine fetal fibroblasts (11% vs. 7%), but the prevalence apoptosis induced in infected fetal fibroblasts transfected with the control vector was relatively low compared to that of the *hIFN-α* transfected cells (77%). This is consistent with the observation that the virus titer was not significantly different between the non-transfected and transfected control groups, but was significantly reduced in the *hIFN-α* transfected cell group. Therefore, P53 may only partially enhance the apoptotic response in virally infected cells [22].

Other IFN-inducible genes such as *PKR* play important roles in promoting apoptosis of virally infected cells [25], [26]. Our qRT-PCR analysis showed that *PKR* expression was significantly induced in the *hIFN-α* transfected cell group, while *PKR* expression was nearly the same between the non-transfected and transfected control groups.

IFN-α has pleiotropic biological effects mediated by hundreds of responsive genes [27]. Therefore, constitutive expression of exogenous IFN-α may have a severely negative impact on a transgenic animal. However, the development of *hIFN-α* transgenic SCNT embryos was not significantly different from that of the control group in our study. Wild-type bovine embryos secrete IFN-τ, a type I IFN, which is an important pregnancy factor known only in ruminants. It has been suggested that extracellular IFN-α elicits the same biologic response as IFN-τ. Both IFN-α and IFN-τ can promote the development of bovine IVF embryos *in vitro* [28], and the growth-promoting effect of hIFN-α was confirmed in our study by adding rhIFN-α-2b to the medium used to culture the bovine IVF embryos (data not shown). However, the intracellular expression of hIFN-α did not significantly promote the development of transgenic SCNT embryos.

This is the first report of the expression of intracelluar IFN-α in embryos. Whether intracelluar IFN-α can elicit the same biological response in embryos as extracellular IFN-α needs to be further investigated. It has been reported that intrauterine application of hIFN-α can change bovine endometrial gene expression in early pregnancy, suggesting that hIFN-α may affect embryo implantation and pregnancy [24]. However, the pregnancy rate of the *hIFN-α* transgenic SCNT embryos was not significantly different from that associated with the control embryos in this study. One explanation for this might be that the non-secreted hIFN-α could not elicit a response from the endometrial cells when transgenic SCNT embryos were implanted into surrogate cows. The two live-born *hIFN-α* transgenic SCNT calves in this study had normal external anatomy and organ development. These results indicate that constitutive expression of intracelluar IFN-α does not have obvious negative effects on the early-stage development of transgenic SCNT calves.

IFN-α can influence the proliferation, differentiation, and function of various types of cells in the immune system and thus influence lympho-hematopoiesis. However, it has been proposed that this effect only takes place under conditions of trauma or inflammation [29]. These factors could explain why expression of hIFN-α did not have obvious adverse effects on transgenic calves in our study. This is supported by a report in which transgenic mice expressing IFN-β (which belongs to the same IFN family as IFN-α) displayed similar behavior, external anatomy, life span, and female fertility as wild type mice, although male transgenic mice displayed reduced fertility [10]. The idiopathic infertilities may be caused by IFNs in the extracellular fluid, which affect the interplay between germ cells and Sertoli cells [22]. It has also been shown that the level of IFN-α in the seminal plasma may be related to sperm production [30]. Intracellular expression of hIFN-α did not result in a high level of IFN-α in the seminal plasma in our study. However, the fertility and the viral resistance of the transgenic calves need to be further determined. The calf is now 17 months old and continued GFP expression has been shown by immunofluorescence in an ear biopsy and in fibroblasts derived from the animal (data not shown).

In conclusion, we constructed a vector with a *hIFN-α* (without secretory signal sequence) gene cassette. Stably transfected bovine fetal fibroblasts were obtained, and *hIFN-α* transgenic embryos were produced by SCNT. Two male cloned calves were born. Expression of intracellular hIFN-α conferred viral resistance on transfected bovine fetal fibroblasts and did not significantly affect the full development of SCNT embryos. These data suggest that *IFN-α* transgenic technology may provide a revolutionary way to achieve disease-control measures through elite breeding of livestock.

## Supporting Information

Figure S1 Schematic of the plasmid pIRESneo-IFN-bCP-LFCIN B-EGFP. The backbone of the vector is pIRSEneo. IFN-NEO cassette: CMV is the promoter of IFN and NEO; LFCIN B gene cassette: goat β-casein regulatory sequence is the promoter of LFCIN B; EGFP gene cassette: CMV is the promoter of EGFP. All restriction sites are shown. Amp, ampicillin resistance gene; IVS, synthetic intron; IRES, internal ribosome entry site of encephalomyocarditis virus; Neo, neomycin phosphotransferase gene; polyA, a fragment of bovine growth hormone poly(A) signal; EGFP, enhanced green fluorescent protein; IFN, β-interferon gene.
(DOCX)
