# Supplementary material for: Expression of Intracellular Interferon-Alpha Confers Antiviral Properties in Transfected Bovine Fetal Fibroblasts and Does Not Affect the Full Development of SCNT Embryos
Source: PLoS One. 2014 Jul 8;9(7):e94444. doi: 10.1371/journal.pone.0094444 (PMC4086716; doi:10.1371/journal.pone.0094444)
Supplement: Figure S1 — Schematic of the plasmid pIRESneo-IFN-bCP-LFCIN B-EGFP. The backbone of the vector is pIRSEneo. IFN-NEO cassette: CMV is the promoter of IFN and NEO; LFCIN B gene cassette: goat β-casein regulatory sequence is the promoter of LFCIN B; EGFP gene cassette: CMV is the promoter of EGFP. All restriction sites are shown. Amp, ampicillin resistance gene; IVS, synthetic intron; IRES, internal ribosome entry site of encephalomyocarditis virus; Neo, neomycin phosphotransferase gene; polyA, a fragment of bovine growth hormone poly(A) signal; EGFP, enhanced green fluorescent protein; IFN, β-interferon gene. (DOCX) [file pone.0094444.s001.docx]

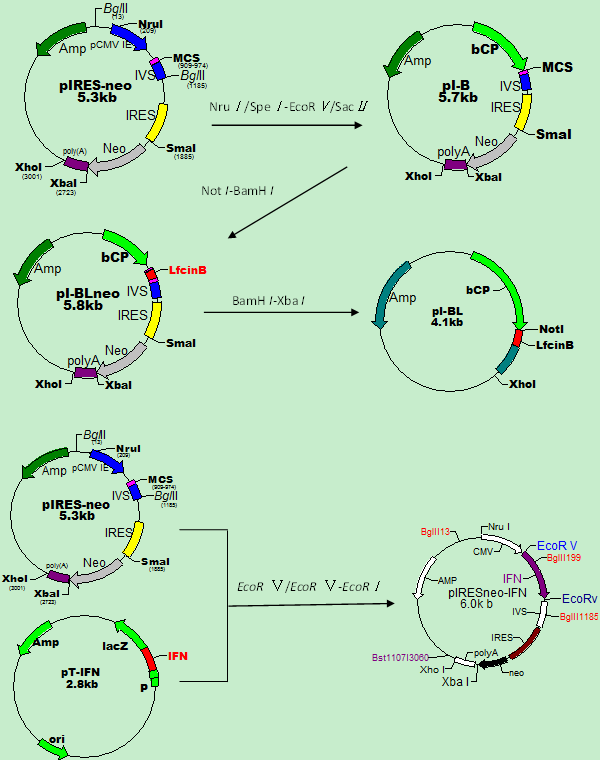


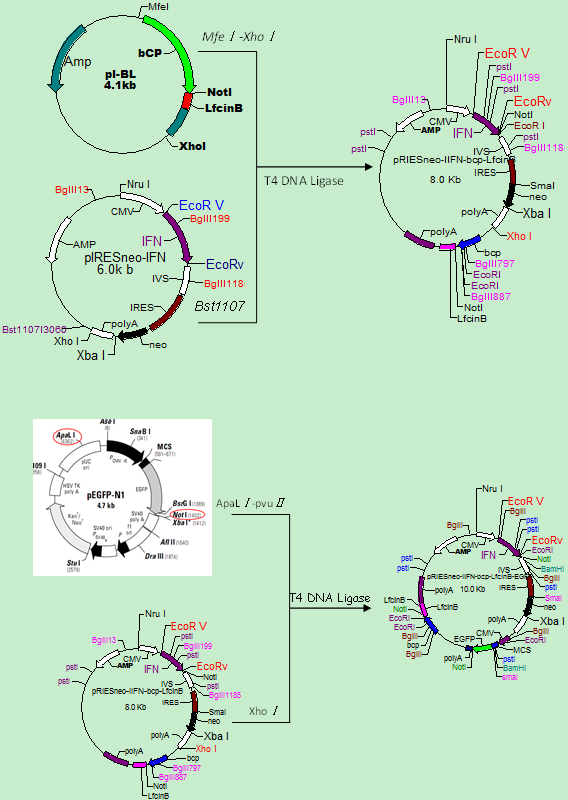


**Figure S1.** **Schematic of the plasmid pIRESneo-*IFN*-bCP-*LFCIN B*-*EGFP*.** The backbone of the vector is pIRSEneo. IFN-NEO cassette: CMV is the promoter of *IFN* and *NEO*; *LFCIN B* gene cassette: goat *β-casein* regulatory sequence is the promoter of *LFCIN B*; *EGFP* gene cassette: CMV is the promoter of *EGFP*. All restriction sites are shown. *Amp*, ampicillin resistance gene; IVS, synthetic intron; IRES, internal ribosome entry site of encephalomyocarditis virus; *Neo*, neomycin phosphotransferase gene; polyA, a fragment of bovine growth hormone poly(A) signal; EGFP, enhanced green fluorescent protein; *IFN*, β-interferon gene.
